# Evolutionary Divergence of Duplicate Copies of the Growth Hormone Gene in Suckers (Actinopterygii: Catostomidae)

**DOI:** 10.3390/ijms11031090

**Published:** 2010-03-16

**Authors:** Henry L. Bart, Paulette C. Reneau, Michael H. Doosey, Charles D. Bell

**Affiliations:** Department of Ecology and Evolutionary Biology, Tulane University, New Orleans, LA 70118, USA; E-Mails: preneau@tulane.edu (P.C.R.); mdoosey@tulane.edu (M.H.D.); cdbell1@uno.edu (C.D.B.)

**Keywords:** cypriniformes, nuclear gene, paralog, parsimony analysis

## Abstract

Catostomid fishes (suckers) have duplicate copies of the growth hormone gene and other nuclear genes, due to a genome duplication event early in the group’s history. Yet, paralogs of GH in suckers are more than 90% conserved in nucleotide (nt) and amino acid (aa) sequence. Within paralogs across species, variation in nt and aa sequence averages 3.33% and 4.46% for GHI, and 3.22% and 2.43% for GHII, respectively. Selection tests suggest that the two GH paralogs are under strong purifying selection. Consensus trees from phylogenetic analysis of GH coding region data for 23 species of suckers, other cypriniform fishes and outgroups resolved cypriniform relationships and relationships among GHI sequences of suckers more or less consistently with analyses based on other molecular data. However, the analysis failed to resolve all sucker GHI and GHII sequences as monophyletic sister groups. This unexpected topology did not differ significantly from topologies constrained to make all GH sequences monophyletic. We attribute this result either to limitations in our GHII data set or convergent adaptive changes in GHII of tribe Catostomini.

## 1. Introduction

Genome duplication has long been thought to play an important role in evolution, giving rise to duplicate copies of genes (paralogs) which subsequently diverge and assume other functions [1,2]. Recent work has highlighted three episodes of genome duplication in vertebrates, which have been linked to the diversification of vertebrates, gnathostomes and teleosts, respectively [3–6]. The three duplication events coincide with bursts of character acquisition and increases in phenotypic complexity in living species, which many researchers attribute to functional divergence of duplicate genes [4]. However, mechanisms of functional divergence are difficult to establish over such long periods of evolutionary time.

Growth hormone (GH) is a single-chain, pituitary-specific hormone essential for promotion and maintenance of somatic growth in vertebrates [7–9]. The GH genomic region in vertebrates is roughly 2 kb long, with the protein coding region divided into four to five blocks (exons) representing less than a third of the length of the genomic region. The GH coding region tends to be highly conserved across vertebrates, presumably because of functional constraints on structure of the hormone. However, rates of GH sequence evolution vary for other groups of vertebrates. GH paralogs in passerine birds were shown to exhibit rapid evolution compared to non-passerines [10]. Comparison of substitution rates in these two groups indicated a 2-fold faster rate of synonymous codon evolution and a 10-fold greater rate of amino acid evolution in passerine birds than in non-passerines. Variability in the rate of evolution of pituitary GH has also been detected in mammals [11]. Whereas GH is highly conserved across most eutherian orders, the gene exhibits 25–50 fold higher rates of evolution in primates and artiodactyls.

Sequences from the GH gene region have been used to infer evolutionary relationships at a variety of taxonomic levels in fishes. GH coding sequences were used to resolve phylogenetic relationships of major clades of fishes [12–16]. Amino acid (aa) sequences from the protein-coding region of GH were first used for inferring the phylogeny of “bony” fishes by Bernardi *et al.* [17]. Interrelationships of major groups of fishes based on GH coding and aa sequences are generally in agreement with relationships based on morphology and other data [12,13,16–19].

GH intron sequences have been used to infer sub-familial phylogenetic relationships of salmonids [20] and labeonines of family Cyprinidae [21], and to characterize intraspecific, population genetic structures of various groups of fishes [8,18,22,23]. GH coding region sequences are being used as part of a multi-gene study of phylogenetic relationships of fishes of Order Cypriniformes [24].

Like salmonids, cypriniform fishes of Family Catostomidae and certain groups of Family Cyprinidae are tetraploids, believed to have arisen due to a hybridization event early in the history of these groups [25]. However, this hypothesis was not tested in an explicitly phylogenetic context, until recently. Work on the GH gene in the catostomid, *Ictiobus bubalus*, has revealed that GH duplication in catostomids was independent of the duplication event that gave rise to paralogous copies of GH in cyprinids [16]. Catostomids are the oldest known cypriniform fishes with fossils dating back to the lower Paleocene, suggesting that the minimum age for the divergence of catostomid species and paralogs of GH is 60 million years.

In this study, we describe genomic organization and size variation of duplicate copies of the GH gene in catostomid fishes. We use GH coding region sequences to infer phylogenetic relationships of paralogous copies of the gene in suckers and other cypriniform fishes. We also use GH coding DNA to infer variation in amino acid composition and structure of the GH protein.

## 2. Results and Discussion

### 2.1. Sequences of Catostomid GH

Partial to complete sequences of two distinct copies of GH were determined for 14 catostomid species; complete sequences for one of the GH copies were obtained from nine additional species (Table 1). BLAST searches of the coding regions revealed high similarity of the new GH sequences with GH copies of *Ictiobus bubalus* and other cypriniform fishes. The two GH copies are named GHI and GHII based on their sequence homology with GH copies in *I. bubalus* [16].

We were able to produce complete coding region data for GHI for most catostomid species using methods described in the experimental section. We were able to produce data for the 5’ end of GHII (Exons 2 and 3) for most catostomid species using GHII specific primers developed in a previous study [16]. However, despite several attempts involving a number of different techniques (also described in the experimental section), thus far we have only been able to produce data for the 3’ end of GHII for species representing tribes Erimyzonini and Catostomini of subfamily Catostominae, in addition to a previously published GHII sequence for *I. bubalus* of subfamily Ictiobinae [ 16].

The genomic organization of GH in suckers is the same as in other Cypriniformes [26,27]. The complete GH genomic sequence comprises five exons and four introns with a total length of 1,500–2,700 nt depending on lengths of the four introns. Exons of different sucker species are of fixed lengths as follows: 10 (Exon 1), 140 (Exon 2), 117 (Exon 3), 162 (Exon 4), and 204 (Exon 5) nt. Introns vary in size across species, from 155–269 (Intron 1), 154–215 (Intron 2), 311–1,188 (Intron 3), and 102–154 (Intron 4) nt (Table 2). The GHII genomic sequence is shorter than that of GHI, with much of the difference due to the substantially longer 3^rd^ intron of GHI.

The GH coding region of catostomids is 633 nt in length. The predicted amino acid (aa) sequences of GHI and GHII encode a protein of 210 aa, which is identical to the protein size reported for other cypriniforms [16,22,28].The putative GH signal peptide cleavage site is serine at aa position 23, which gives a predicted mature polypeptide size of 188 aa, consistent with other cypriniform species [28].

The two GH copies are very similar in both nt and aa sequence composition. Mean nt divergence between GHI and GHII is 9.61%. Mean pairwise aa sequence divergence between copies is 8.53%. Mean pairwise nt sequence divergence within paralogs (coding region data only) across catostomid species is 3.33% for GHI and 3.22% for GHII. Mean aa divergence within paralogs is 4.46% for GHI and 2.43% for GHII. The lower percentage in aa divergence for GHII is due to the incomplete data for several of the catostomine species.

An interesting and potentially evolutionarily significant difference in GH copies of suckers involves variation in the number of cysteine residues in the mature peptide. Pairs of cysteine residues form disulfide bonds, important to protein folding and stability [29]. GH in all vertebrates has four cysteine residues in highly conserved positions in the amino acid sequence. Ostariophysan fishes have an unpaired, fifth cysteine in aa position 145. In GHI of catostomids, the extra cysteine is replaced by tyrosine. The functional significance of this disparity has yet to be established.

### 2.2. Phylogenetic Analysis of GHI and GHII

**Figure 1.**
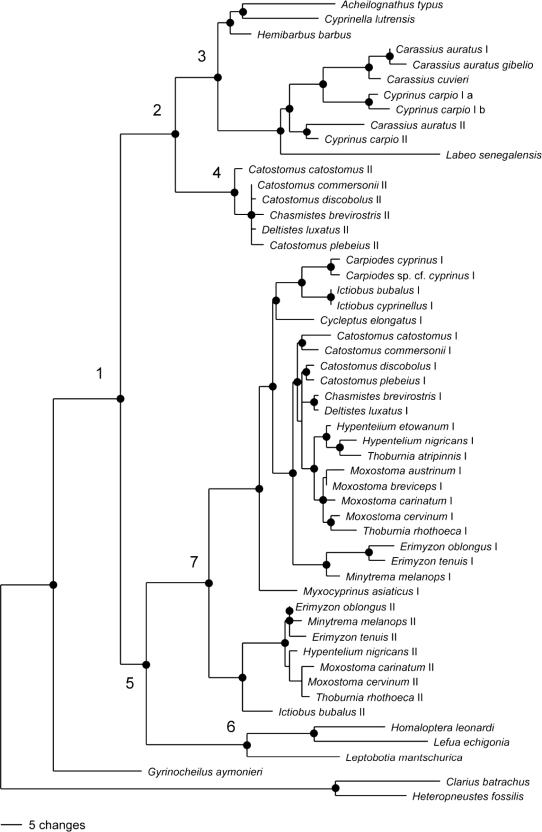
Majority rule consensus tree of 2000 bootstrap replicates from maximum parsimony analysis of cypriniform GH sequence data. Filled circles at nodes represent bootstrap support greater than 95%.

The consensus trees obtained with MP and ML analyses are identical. Only the MP tree is shown (Figure 1). The MP analysis is based on 230 parsimony informative sites in the combined GHI/GHII data set (300 sites are constant). The MP consensus tree is 726 steps long. Order Cypriniformes (Node 1 in Figure 1) is recovered as a monophyletic group with strong bootstrap support. *Gyrinocheilus aymonieri* (Family Gyrinocheilidae) is strongly supported as the most basal cypriniform. Thus, GH data does not support a monophyletic Superfamily Cobitoidea inclusive of gyrinocheilids, loaches and catostomids, as supported by morphology [30,31] and analysis of multiple nuclear genes and mitogenome data [24].

Two strongly supported interfamilial groups make up the strongly supported sister group to *Gyrinocheilus aymonieri*. The first of these groups (Node 2) comprises a strongly supported family Cyprinidae (Node 3), sister to a strongly supported group of GHII sequences for representatives of tribe Catostomini (Node 4). The second group (Node 5) comprises a strongly supported basal group of cobitids and balitorids (Node 6), sister to a strongly supported group of GHI and GHII sequences representing other subfamilies and tribes of family Catostomidae (Node 7).

Family Cyprinidae comprises strongly supported subfamily groups of Cyprinines, and Leuciscines plus Gobionines. Within subfamily Cyprininae, the two copies of GH in tribe Cyprinini form a strongly supported monophyletic group, with sequences for each of the copies forming strongly supported monophyletic sister groups.

Five nt substitutions link cyprinid GH sequences with GHII sequences of suckers representing tribe Catostomini, thus rendering the two copies of GH in suckers, and catostomids as a whole, non monophyletic. In contrast, the GHI portion of the tree is well-resolved, monophyletic, and more or less consistent with hypotheses of catostomid relationships based on other data [32,33]. GHI of *Myxocyprinus asiaticus* is most basal. This species is sister to a strongly supported group comprising a monophyletic Catostominae GHI plus a strongly supported group of *Cycleptus elongatus* plus a monophyletic subfamily Ictiobinae GHI, the latter group comprising a monophyletic *Carpiodes* plus a monophyletic *Ictiobus*. The catostomid GHI tree is the strongly supported sister group to GHII sequences for the remaining catostomid species. In the latter group, *Ictiobus bubalus* GHII is basal and sister to a strongly supported group comprising GHII sequences for species representing tribes Erimyzonini, Moxostomatini and Thoburniini of subfamily Catostominae.

The sister group relationship of Catostomini GHII sequences with cyprinids was unexpected. Of the five nt substitutions inferred along this branch, four are not shared with other catostomid GH sequences, and two of these substitutions result in aa changes that are also not shared with other catostomids (valine to methionine at aa position 90 and leucine to methionine at aa position 169). The two aa substitutions are in C-terminal end of the protein, corresponding to Exons 4 and 5. GHII data from this end of the gene is available only for *Minytrema melanops* among Tribes Erimyzonini, Moxostomatini and Thoburniini. GHII sequences of Tribe Catostomini share nine nt characters with GHI and/or GHII sequences from other catostomids and would likely share more if GHII data were more complete. Two of the nine substitutions result in aa changes that are convergent with aa character states in other catostomid GH sequences (serine to cysteine in aa position 14 [signal peptide] and glycine to aspartic acid in aa position 81). It is possible that missing GHII data from the 3’ end of the gene, especially for other tribes of catostomines, would have supported a different tree topology.

When all catostomid GHII sequences are constrained to be monophyletic, the resulting tree is 11 steps longer than the MP consensus tree. When Catostomini GHII sequences are constrained to be the sister group of catostomid GHI plus the remaining GHII sequences, the resulting tree is only four steps longer than the MP consensus tree. Based on Templeton test results, neither constraint tree is significantly longer than the MP consensus tree (GHII monophyletic: Z = −1.9149, p = 0.0555; Catostomini GHII sister to remaining catostomid GHI and GHII sequences: Z= − 0.8944, p = 0.5034).

### 2.3. Selection Tests

We compared coding sequences of the mature GHI and GHII proteins of catostomids to gain insight into the possible evolutionary forces affecting the divergence of the two copies of the hormone. The comparison revealed a lower number of non-synonymous differences per non-synonymous site (*d*_N_) relative to the number of synonymous differences per synonymous site (*d*_S_) (*P* = 0.003, Z-test of positive selection), indicating a paucity of amino acid replacement changes compared with neutral expectations. Thus, the null hypothesis of strict neutrality (*d*_N_ = *d*_S_) can be rejected in favor of the alternative hypothesis of purifying selection (*d*_N_ < *d*_S_) for all catostomid species. Purifying selection is also suggested for pairwise comparisons of GHI and GHII of the cyprinids *Carassius auratus* and *Cyprinus carpio*. There is no evidence for positive selection among the GH sequences tested. The slow rate of divergence of the GH coding region observed across suckers and other cypriniforms is not surprising considering the protein’s critical role in promoting growth and differentiation at distant target sites [34] as well as its secondary functions in autocrine/paracrine regulation of cellular differentiation during embryonic development [35,36].

## 3. Experimental Section

### 3.1. DNA Extraction, PCR, and Sequencing

Total DNA was extracted from ethanol preserved muscle or fin tissue with the Purelink Genomic DNA Mini Kit (Invitrogen, Carlsbad, CA). PCR amplification was conducted in two steps, long PCR and full-nested short PCR. The long PCR primer pair GH22F (5′-YTGTCKDTGGTSCTGGTYAGT-3′) and GHR (5′-CAGGGTRCAGTTKGAATCSAR-3′) was used in a 15.5-μL reaction mixture containing 9.725 μL sterile water, 1.5 μL Ex Taq buffer, 1.2 μL dNTPs (2.5mM), 1.0 μL each primer (10 μM), 0.075 μL *Taq* polymerase (Takara *Ex Taq*, Takara, Japan), and 1.0 μL of template DNA (ca. 50 ng/μL). The thermal cycle protocol was as follows: (1) initial denaturation at 94 °C for 60 s; (2) then 30 cycles of denaturation at 94 °C for 30 s; annealing at 52 °C for 30 s; and extension at 72 °C for 120 s; and (3) final extension at 72 °C for 10 min. The first round PCR produced an amplicon ranging from 1,200 to 2,300 bp (depending on length of introns) that spans half of exon 2 to near the termination codon in exon 5. This product contained both GH copies and was cloned in the pGEM-T Easy Vector (Promega, Madison, WI). Positive colonies (*i.e.* white colonies) were selected and used as a template for short PCR. Short PCR was conducted using up to three internal primer pairs GH22F and GH264R (5′-GCTYTTYTGBGTTTCATSTTT-3′), GH181F (5′-CAGCTGAGTAAAATCTTYCCT-3′) and GH295R (5′-CTCCCARGAYTCAATGAGGYG-3′), and GH274F (5′-AAGCTBCTTCGYATCTCYTT-3′) and GHR with the same reaction mixtures as above [16,24]. Thermal cycling profile was the same as first round PCR except extension time was reduced to 30 s. Part of the 5’ UTR and exons 1 through 3 were amplified and sequenced for catostomids by pairing GH copy specific primers GHIF (5′-AAAGCCTTCAACTAAGACTAAC-3′) and GHIIF (5′-CAAACCTTCAACTAAGACTTCA-3′), developed for *Ictiobus bubalus* [24], with primer GH240R (5′-TTCTGGGTTTCATGTTTGTCA-3′). Short PCR products were purified with ExoSAP-IT (USB, Cleveland, OH) and directly sequenced using BigDye Terminator v3.1 Cycle Sequencing Kits (Applied Biosystems, Carlsbad, CA). The resulting products were analyzed on an ABI 3730*xl* Genetic Analyzer (Applied Biosystems).

Obtaining complete coding region sequences of GHII for all sucker species proved challenging because we were not able to design internal primers specific to the 3’ end of this gene copy. We tried designing primers specific to the different sucker tribes and nesting them with our GHII-specific upstream primer and a non-specific downstream primer. This amplified both GH copies. We varied PCR techniques to increase GH yield by performing re-extensions, reconditioning PCR, and varying primer and DNA volumes. This resulted in non-specific primer binding with multiple bands observed during gel electrophoresis. We extended electrophoresis runs on PCR products, cutting out and gel purifying double bands and cloning both products. This yielded large sequences of GHI, but very small fragments of GHII. Lastly, we diluted the ligation mix during cloning in an effort to decrease plasmid incompatibility, thereby increasing the cloning efficiency of paralogous sequences. This method yielded the 3’ GHII data for species we have completed thus far.

### 3.2. Sequence Alignment,Variation, and Phylogenetic Analysis

Sequence chromatograms were assembled into contigs and edited with Sequencher 4.6 (Gene Codes, Madison, WI). Inconsistencies in base calls in cloned fragments were infrequent and were resolved by simple majority or left ambiguous. Additional GH sequences were obtained from NCBI by taxonomy and BLASTN searches. Sequences were aligned using CLUSTAL W [37] as implemented in BioEdit [38] and visually inspected for errors and improved manually. Sequence divergence (Tamura-Nei distance), Maximum Likelihood (RAxML [39]) and Maximum Parsimony (PAUP* [40]) analyses were performed on GH coding region data only, using the CIPRES web portal (www.phylo.org). Node support is based on 2,000 bootstrap replicates. The extent of nucleotide sequence divergence was estimated by means of the uncorrected differences (*p* distance). Sequence variation was examined by plotting pairwise transitional (TS) and transversional (TV) differences against *p* distance.

Templeton tests, implemented in PAUP* [40], were conducted to test for differences in the lengths of the MP consensus tree and two alternative topologies constrained as follows: 1) All catostomid GHII sequences monophyletic; 2) Catostomini GHII sister to remaining catostomid GHI and GHII sequences.

### 3.3. Selection Tests

The number of synonymous substitutions per synonymous site (*d*_S_) and nonsynonymous substitutions per nonsynonymous site (*d*_N_) used in selection tests were estimated using the method of Nei and Gojobori [41] as implemented in *MEGA* version 4 [42]. Nucleotide and amino acid distances were estimated using a pairwise deletion option for each catostomid species for which complete or partial GHI and GHII sequence were determined. The presence of positive selection was analyzed by testing the null hypothesis that H*_o_*: *d*_N_ = *d*_S_, versus the alternative positive selection hypothesis that H1: *d*_N_ > *d*_S_ using the codon based z-test for selection [43]. The z-statistic and the probability that the null hypothesis is rejected were obtained as indicated by P > 0.05.

## 4. Conclusions

Suckers possess two copies of the growth hormone gene, presumably as a result of a genome duplication event early in the family’s history. The two gene copies are remarkably similar in both coding region nt sequence and aa sequence composition (>90% sequence homology) considering the antiquity of Family Catostomidae. Both GH copies have four cysteine residues in highly conserved positions in the amino acid sequence, which are common to all vertebrates. GHII has a fifth cysteine residue in aa position 145, which is common to all ostariophysan fishes. In GHI of catostomids, the fifth cysteine is replaced by tyrosine. The functional significance of this disparity has yet to be established.

The genomic organization of GH in suckers is the same as in other Cypriniformes, comprising five exons and four introns with a total length of 1,500–2,700 nt, depending on lengths of the four introns. The GHII sequence is shorter than GHI, with much of the difference due to the substantially longer 3^rd^ intron of GHI. An important limitation of this study is that we were only able to produce data for the 5’ end of GHII (Exons 2 and 3) for several species representing tribes Erimyzonini and Moxostomatini of Subfamily Catostominae.

The pattern of phylogenetic relationships among cypriniform fishes inferred from coding region sequences of the nuclear GH gene agrees in most respects with relationships inferred from other molecular data. The patterns of relationships among suckers inferred from sequences of GHI and a subset of the GHII sequences are consistent and in basic agreement with relationships based on other data. The only unusual result is the sister relationship between GHII sequences of Tribe Catostomini and cyprinid GH sequences. Although this topology is not significantly different from topologies constrained to make all catostomid GHI and GHII sequences monophyletic, it is the most parsimonious topology and it is supported by uniquely derived nt and aa characters. There are two possible explanations for this result, both requiring additional study: (1) it reflects the effects of incomplete GHII data for a number of catostomine species on character state reconstruction in this portion of the GHII tree; (2) it reflects homoplasy resulting from purifying selection or other functional constraints on GHII evolution. We are gathering the necessary data to address the first of these possibilities before addressing the second.

## Figures and Tables

**Table 1.** List of species used in the comparative and phylogenetic analysis of catostomid GH sequences.

**Species**	**Copy**	**Source**	**GenBank number**
*Carpiodes cyprinus*	I	this study	GU937834
*Carpiodes sp.* cf. *cyprinus*	I	this study	GU937849
*Catostomus catostomus*	I	this study	GU937824
*C. catostomus*	II	this study	GU937826
*C. commersonii*	I	Mayden *et al.* 2009	FJ265027
*C. commersonii*	II	this study	GU937823
*C. discobolus*	I	this study	GU937830
*C. discobolus*	II	this study	GU937832
*C. plebeius*	I	this study	GU937833
*C. plebeius*	II	this study	GU937829
*Chasmistes brevirostris*	I	this study	GU937825
*C. brevirostris*	II	this study	GU937827
*Cycleptus elongatus*	I	Mayden *et al.* 2009	FJ265028
*Deltistes luxatus*	I	this study	GU937831
*D. luxatus*	II	this study	GU937828
*Erimyzon oblongus*	I	this study	GU937837
*E. oblongus*	II	this study	GU945705
*E. tenuis*	I	this study	GU937838
*E. tenuis*	II	this study	GU937839
*Hypentelium etowanum*	I	this study	GU937836
*H. nigricans*	I	Mayden *et al.* 2009	FJ265055
*Ictiobus bubalus*	I	Clements *et al.* 2004	AY375301
*I. bubalus*	II	Clements *et al.* 2004	AY375302
*I. cyprinellus*	I	this study	GU937840
*Minytrema melanops*	I	Mayden *et al.* 2009	FJ265050
*M. melanops*	II	this study	GU937822
*Moxostoma austrinum*	I	this study	GU937841
*M. breviceps*	I	this study	GU937842
*M. carinatum*	I	this study	GU937843
*M. carinatum*	II	this study	GU937835
*M. cervinum*	I	this study	GU937844
*M. cervinum*	II	this study	GU937845
*Myxocyprinus asiaticus*	I	Mayden *et al.* 2009	FJ265052
*Thoburnia atripinnis*	I	this study	GU937846
*T. rhothoeca*	I	this study	GU937847
*T. rhothoeca*	II	this study	GU937848
*Acheilognathus typus*		Mayden *et al.* 2009	FJ265056
*Carassius auratus*	I	Law *et al.* 1996	AF069398
*C. auratus*	II	Law *et al.* 1996	AF069399
*C. a. gibelio*		unpublished	AY265352
*Clarius batrachus*		unpublished	AF416485
*Cyprinella lutrensis*		Mayden *et al.* 2009	FJ265061
*Cyprinus carpio*	I a	Mayden *et al.* 2009	FJ265047
*C. carpio*	I b	unpublished	AJ640136
*C. carpio*	II	unpublished	AJ640135
*Gyrinocheilus aymonieri*		Mayden *et al.* 2009	FJ265031
*Hemibarbus barbus*		Mayden *et al.* 2009	FJ265032
*Heteropneustus fossilis*		unpublished	AF416489
*Homaloptera leonardi*		Mayden *et al.* 2009	FJ265022
*Labeo senegalensis*		Mayden *et al.* 2009	FJ265034
*Lefua echigonia*		Mayden *et al.* 2009	FJ265023
*Leptobotia mantschurica*		Mayden *et al.* 2009	FJ265035

**Table 2.** Genomic organization of GH gene in suckers includes length of UTRs, introns, GH fragment sequenced (Gene), and coding sequence (CDS). Museum vouchers are included when available.

**Species**	**Copy**	**Voucher**	**5′ UTR**	**Intron 1**	**Intron 2**	**Intron 3**	**Intron 4**	**3′ UTR**	**Gene**	**CDS**

*Carpiodes cyprinus*	I	None		na	na	na	na			607
*Carpiodes* sp. cf. *cyprinus*	I	None			207	985	154		1878	532
*Catostomus catostomus*	I	UAIC 11218.05		228	194	949	154		2117	550
*C. catostomus*	II	UAIC 11218.05	39	174	194	311	145		1470	607
*C. commersonii*	I	None	47	235	175	592	102		1788	633
*C. commersonii*	II	None	31	222	194	311	145		1513	610
*C. discobolus*	I	BYU 57986	36	235	198	599	102		1711	541
*C. discobolus*	II	BYU 57986	48	220	194	311	142		1531	613
*C. plebeius*	I	MSB 49632		235	198	599	102		1683	542
*C. plebeius*	II	MSB 49632		220	194	311	145		1512	603
*Chasmistes brevirostris*	I	OS 15963	35	235	198	596	102		2037	633
*C. brevirostris*	II	OS 15963		210	194	311	145		1492	593
*Cycleptus elongates*	I	TU 192331		242	203	965	143		2125	571
*Deltistes luxatus*	I	OS 15922	35	236	198	596	102		1925	633
*D. luxatus*	II	OS 15922		210	194	311	145		1517	611
*Erimyzon oblongus*	I	NCSM 37439			185	955	154		1845	543
*E. oblongus*	II	NCSM 37439	557	227	199				1157	150
*E. tenuis*	I	None	259	259	192	947	154		2395	633
*E. tenuis*	II	None	139	225	199				826	263
*Hypentelium etowanum*	I	None	176	248	197	832	146		2293	633
*H.nigricans*	I	None	147	252	197	901	146		2212	569
*H. nigricans*	II	None	639	226	181				1287	225
*Ictiobus bubalus*	I	TU 196158	56	Na	na	na	na	590		633
*I.bubalus*	II	TU 196158	56	Na	na	na	na	590		633
*I. cyprinellus*	I	None			204	870	154		1787	559
*Minytrema melanops*	I	TU 193988	147	254	193	1188	154		2569	633
*M. melanops*	II	TU 193988	627	225	182	320^a^	154		2141^a^	633
*Moxostoma austrinus*	I	None		261	200	914	143		2074	548
*M. breviceps*	I	None	38	225	200	936	143		2084	542
*M. carinatum*	I	None	211	259	200	936	143		2392	633
*M. carinatum*	II	None		225	179					150
*M. cervinum*	I	None		263	200	928	143		2092	542
*M. cervinum*	II	None	618	224	181					267
*Myxocyprinus asiaticus*	I	None	31	269	215	969	154		2180	541
*Thoburnia atripinnis*	I	None	211	252	195	936	152		2442	633
*T. rhothoeca*	I	None	208	252	199	914	146	64	2416	633
*T. rhothoeca*	II	None	638	224	154					267
